# Multi‐omics analysis reveals multiple mechanisms causing Prader–Willi like syndrome in a family with a X;15 translocation

**DOI:** 10.1002/humu.24440

**Published:** 2022-07-23

**Authors:** Jesper Eisfeldt, Fatemah Rezayee, Maria Pettersson, Kristina Lagerstedt, Helena Malmgren, Anna Falk, Giedre Grigelioniene, Anna Lindstrand

**Affiliations:** ^1^ Department of Molecular Medicine and Surgery Karolinska Institutet Solna Sweden; ^2^ Department of Clinical Genetics Karolinska University Hospital Stockholm Sweden; ^3^ Science for Life Laboratory Karolinska Institutet Science Park Solna Sweden; ^4^ Department of Neuroscience Karolinska Institute Stockholm Sweden

**Keywords:** chromosomal translocation, iPSC, multi‐omics, neuroepithelial stem cells, Prader–Willi syndrome, RNA sequencing, trisomy rescue, whole‐genome sequencing

## Abstract

Prader–Willi syndrome (PWS; MIM# 176270) is a neurodevelopmental disorder caused by the loss of expression of paternally imprinted genes within the PWS region located on 15q11.2. It is usually caused by either maternal uniparental disomy of chromosome 15 (UPD15) or 15q11.2 recurrent deletion(s). Here, we report a healthy carrier of a balanced X;15 translocation and her two daughters, both with the karyotype 45,X,der(X)t(X;15)(p22;q11.2),−15. Both daughters display symptoms consistent with haploinsufficiency of the *SHOX* gene and PWS. We explored the architecture of the derivative chromosomes and investigated effects on gene expression in patient‐derived neural cells. First, a multiplex ligation‐dependent probe amplification methylation assay was used to determine the methylation status of the PWS‐region revealing maternal UPD15 in daughter 2, explaining her clinical symptoms. Next, short read whole genome sequencing and 10X genomics linked read sequencing was used to pinpoint the exact breakpoints of the translocation. Finally, we performed transcriptome sequencing on neuroepithelial stem cells from the mother and from daughter 1 and observed biallelic expression of genes in the PWS region (including *SNRPN*) in daughter 1. In summary, our multi‐omics analysis highlights two different PWS mechanisms in one family and provide an example of how structural variation can affect imprinting through long‐range interactions.

## 1 BACKGROUND

Prader–Willi syndrome (PWS; MIM# 176270) is a complex disorder, affecting 1 out of 10,000 individuals (Butler et al., 2019; Cassidy et al., 2012). PWS is associated with a wide range of symptoms that varies across ages and patients; the characteristic symptoms include failure to thrive and feeding difficulties in newborn, developmental delay and obesity in children, as well as obesity and hypogonadism in adults (Miller et al., 2011). As of date, there is no cure for the patients affected by PWS, however, there are various treatments that may lessen the symptoms (Butler et al., 2019).

PWS is caused by loss of expression of the paternal copy of 15q11.2. Such loss of expression may be caused by deletion (roughly 60% of cases), maternal uniparental disomy of chromosome 15 (UPD15) (35% of cases), as well as imprinting mutations (Butler et al., 2019).

Multiple genes are located in the PWS imprinted region on 15q11.2, that are expressed exclusively from the paternal copy of the region, including *MKRN3*, *MAGEL2*, *NDN*, *SNURF‐SNRPN*, *PWRN1*, *PWRN2*, and *IPW* (Cheon, 2016). It is not known how these genes contribute to PWS, and it is believed that different genes contribute to different traits of the phenotype, explaining the phenotypic variability of the PWS patients (Nicholls et al., 1989). Notably, changes to the 15q11.2 region may cause Angelman syndrome, caused by the loss of expression of the maternally inherited 15q11.2 region.

The PWS critical region (PWCR) has been extensively studied, partly due to the relatively high frequency of PWS and Angelman syndrome. The genes residing within the PWS region have been interrogated through a multitude of studies, including from case reports (Nicholls et al., 1989), animal studies (Bervini & Herzog, 2013), and cell cultures (Chamberlain et al., 2010). Despite these efforts, the interplay and function of these genes remains unknown.

Carriers of balanced chromosome aberrations (1:500) are usually healthy, but due to errors in meiotic recombination and malsegregation of the rearranged chromosomes, they have a risk (Warburton, 1991) of recurrent abortions and having children with unbalanced rearrangements. Moreover, if the chromosomal breakpoints disrupt or dysregulate important genes they can result in disease. Furthermore, even when translocation breakpoints are located outside of genes, the disruption of the DNA 3D structure may lead to altered gene expression. One important example of this phenomena are the topologically associated domains (TADs) (Lupiáñez et al., 2015), regions of DNA with a high degree of self‐interactivity that are isolated from other genomic regions by TAD boundaries (McArthur & Capra, 2021). A number of publications have shown that such disruptions may lead to abberant gene expression and various human diseases.

Herein, we present a familial X;15 translocation, including a healthy carrier of the balanced form and her two daughters, both affected by PWS, with the same unbalanced karyotype (45,X,der(X)t(X;15)(p22;q11),−15). By applying a combination of traditional cytogenetics and molecular genetic investigations with multi‐omics profiling of patient‐derived stem cells, we show that different mechanisms are responsible for PWS in the two daughters.

## 2 METHODS

### 2.1 Ethics approval and consent to participate

The Regional Ethical Review Board in Stockholm, Sweden approved the study (Genomic studies, Dnr79 2012/2106‐31/4 and cellular studies, Dnr 2016/430‐31). Written informed consent was obtained.

### 2.2 Clinical synopsis

Daughter 1 had a normal birth weight at term (3.885 kg, +1.1 SD), birth length (52 cm, +1.0 SD). At 4 years 11 months, her height was 106.9 cm (−0.9 SD) and weight 25.7 kg (+2.2 SD) (BMI 22.49, target height −0.8) (Albertsson‐Wikland et al., 2020). She was treated with growth hormone from 9 years 9 months for 1 year with high dose (0.043 mg as highest) with minimal effect on height development. At 18 years, she is 154 cm (−2.0 SD) and her weight is 100 kg (+4.0 SD) (BMI 42.17). At age 10, she presented with mild Madelung deformity. She has mild cognitive difficulties diagnosed in young adult age.

Daughter 2 was born at term and showed severe muscular hypotonia at birth (Apgar scores 6, 7, 8) and was diagnosed with PWS within 1 month. Her birth weight was normal (3.338 g, −0.1SD), birth length (49.7 cm, 0 SD). A small ventricular septal defect without hemodynamic consequences was present. Her early symptoms of PWS were mild and she did not need gastric tube feeding. At 11 months, she was 71.2 cm (−0.5 SD) and 9.08 kg (−0.2 SD). She was treated with growth hormone from 3 years of age. Her final height at age 18 is 154 cm (−2.0 SD) and 66.9 kg (+0.8 SD). From an early age, she had developmental delay and symptoms consistent with autism. She did not have Madelung deformity at 12 years.

### 2.3 Chromosome analyses

Metaphase slides were prepared from peripheral blood cultures according to standardized protocols. Chromosome analysis was performed according to routine procedures with the GTG‐banding technique and an approximate resolution of 550 bands per haploid genome was obtained.

### 2.4 Fluorescent in situ hybridization (FISH)‐mapping

Breakpoint mapping was performed by FISH with human genomic bacteria artificial chromosomes (BACs) mapping to the translocation breakpoint regions on human chromosomes X and 15 (www.ensembl.org). In brief, BACs were ordered from The Wellcome Trust Sanger Institute or BACPAC Resource Center Children's Hospital (Oakland Research Institute) and delivered as bacterial luria broth agar stab cultures. The clones were isolated, prepared, and labeled as described previously (Malmgren et al., 2007). At least 10 metaphases per hybridization were analyzed.

### 2.5 Methylation assay and UPD‐analysis

Genomic DNA from the mother and her two daughters were analyzed using multiplex ligation‐dependent probe amplification (MLPA) using the methylation sensitive diagnostic MLPA kit ME028 (MRC Holland), using the instructions from the provider. The MLPA‐products were analyzed on an ABI3500xL instrument (Applied Biosystems). Electropherograms were analyzed using GeneMarker software (SoftGenetics LLC). The obtained normalized quotients for the different probes were considered a deletion when below 0.75 and duplication when above 1.3. Regarding the methylation specific probes (sensitive to Hha1 digestion), deviation in methylation were considered when the normalized quotients was above 1.7.

UPD was verified analyzing microsatellite markers surrounding the *SNRP* gene (D15S646, D15S128, D15S1513, and D15S97). polymerase chain reaction (PCR) products were analyzed on an ABI3500xL instrument (Applied Biosystems). Electropherograms were analyzed using GeneMapper 6 (Applied Biosystems).

### 2.6 Short‐read sequencing

Genomic DNA derived from whole blood from daughter 1 was sequenced using two distinct Illumina WGS protocols: 30X PCR‐free paired‐end (PE) protocol at National Genomics Infrastructure (NGI), Stockholm, Sweden. Data were processed and analyzed as described previously (Eisfeldt et al., 2019). Briefly, data were pre‐processed using the NGI‐piper pipeline (https://github.com/NationalGenomicsInfrastructure/piper) and structural variants were called using the FindSV (https://github.com/J35P312/FindSV) pipeline that combines CNVnator (Abyzov et al., 2011) and TIDDIT (Eisfeldt et al., 2017). Variants of interest were visualized in Integrative Genomics Viewer (IGV) (Thorvaldsdóttir et al., 2013).

### 2.7 Linked‐read sequencing

Genomic DNA derived from whole blood from the unaffected mother was sequenced using the 10X Genomics Chromium WGS protocol and data were analyzed and processed as described previously (Eisfeldt et al., 2019). Data were analyzed using 10X Genomics default pipelines Long Ranger V2.1.2 (https://support.10xgenomics.com/genome-exome/software/downloads/latest).

### 2.8 Neuroepithelial stem (NES) cell cultivation and transcriptome sequencing

NES cells were grown at the Karolinska Institutet iPS Core facility, according to their standard protocols (https://ipscore.se/se/). NES cells were produced from iPS cells derived from fibroblasts, sampled from the unaffected mother, daughter 1, as well as three healthy unrelated controls. Transcriptome sequencing was performed on each of these five individuals. The transcriptome sequencing was performed at NGI Stockholm, using a Ribozero‐prep, sequencing 44 million 150 bp paired‐reads per replicate on the Nova‐seq platform. The resulting data was aligned to hg19 using STAR and Salmon (Patro et al., 2017). Single nucleotide variants (SNVs) were called using the GATK haplotype caller (van der Auwera et al., 2013), and differential expression analysis was performed using Deseq2 (Love et al., 2014).

### 2.9 Droplet digital PCR

In brief, complementary DNA (cDNA) was prepared from fibroblasts as well as from NES cells. The droplet digital PCR (ddPCR) assays (primers and probes) were designed targeting one known SNV located within SNRPN gene and the corresponding wildtype. The experiments were performed on QX200 AutoDG Droplet Digital PCR System/QX200 Droplet Reader (BioRad) according to the manufacturer's recommendations. Obtained data were analyzed using the QuantaSoftPro/QX Manager Software (BioRad) and the ratio between SNV target and wildtype were calculated for each sample.

### 2.10 TAD analysis

Bed files describing the positioning of TADs in cortex, thymus, small bowel, and lung were downloaded from (http://3dgenome.fsm.northwestern.edu/) (Wang et al., 2018). The bed files were indexed and searched using TABIX (Li, 2011), and visualized using IGV (Thorvaldsdóttir et al., 2013).

## 3 RESULTS

### 3.1 Cytogenomic studies

**Figure 1:**
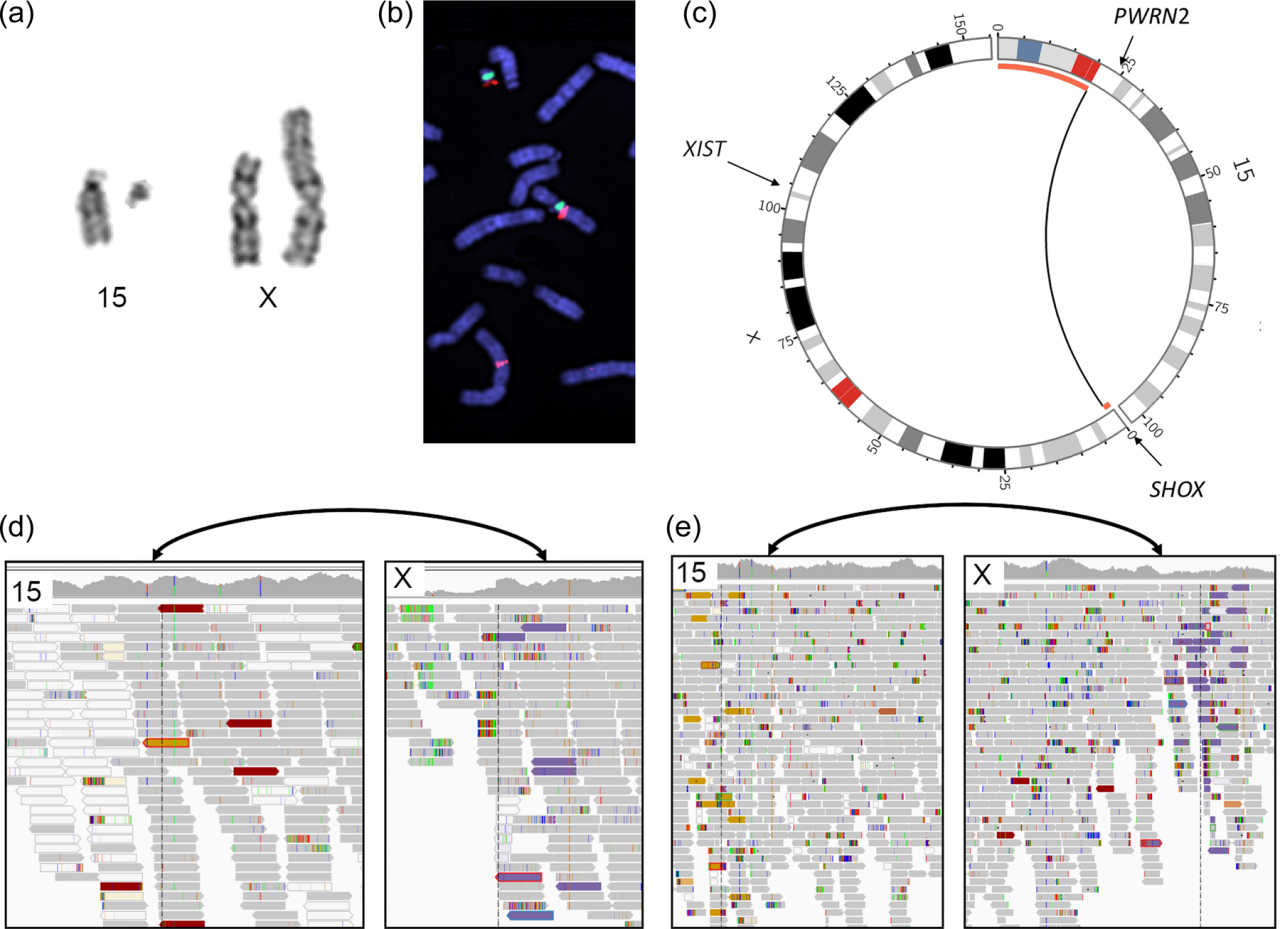
Chromosome analyses using cytogenetics and WGS. (a) The karyotype of the balanced X;15 translocation. (b) FISH mapping of metaphases from the mother showing BAC RP11‐622H13 split between 15q11.2 and Xp22.33. A probe specific to Centromere 15 is shown in green. (c) Circos plots illustrating the familial translocation, the black arc indicates the breakpoint positions and the red bars indicate regions deleted in the daughters due to unbalanced segregation. (d) and (e) IGV screenshots illustrating the breakpoint regions in daughter 1 (short read WGS) (d), and unaffected mother (linked read WGS) (e). For both (d) and (e), a bidirectional arrow in black indicate the location of the breakpoints and the discordant read pairs pinpointing the translocation are highlighted in gold (chr15) and purple (chrX). FISH, fluorescent in situ hybridization; IGV, Integrative Genomics Viewer.

Chromosome analysis revealed a de novo balanced translocation between chromosomes 15 and X (46,XX,t(X;15)(p22.3;q11.2)) in the unaffected mother (Figure 1a,b), and follow‐up studies revealed a derivative translocation, 45,X,der(X)t(X:15)(p22.3;q11.2),−15, in both daughters, inherited in an unbalanced manner (Figure 1c). Microsatellite markers indicated UPD15 in daughter 2 but not in daughter 1. Methylation sensitive MLPA showed aberrant *SNRPN* signals consistent with PWS in daughter 2 and normal patterns in daughter 1 and the mother (Table 1).

**Table 1:** Summary of the analysis performed

Case	Mother	Daughter 1	Daughter 2
Cytogenic	46,XX,t(X;15)(p22.3;q11.2)	45,X,der(X)t(X:15)(p22.3;q11.2),−15	45,X,der(X)t(X:15)(p22.3;q11.2),−15
MLPA chr15	Normal	Normal	Maternal UPD15
SR‐WGS	NA	Not detected	NA
lrWGS	46,XX,t(X;15)(p22.3;q11.2) NC_0000023.10:g.pter_1517433delins[NC_000015.9:g. 20641578_pterinv]	NA	NA
	NC_0000023.10g.qter_15174334invdelins[NC_000015.9:g. 20641579_qter]		
RNA‐seq	Monoallelic *SNRPN*	Biallaelic *SNRPN*	NA
droplet digital PCR	Monoallelic *SNRPN*	Biallaelic *SNRPN*	Monoallelic *SNRPN*

FISH mapping could pinpoint the genomic breakpoint region to within clones RP11‐622H13 on chromosome 15q11.2, chr15: 20487047‐20627801, and RP11‐261P4 on chromosome Xp22.33, chrX:1497956‐1660348.

Genomic characterization was first done by short read WGS of daughter 1; however, the translocation was not detected by our pipeline or manual inspection (Figure 1d). Next, the mother was sequenced using linked‐read WGS, revealing the exact breakpoint positions of the translocation (chr15:20641579, chrX:1517434) matching the FISH mapping results (Figure 1e). The chromosome 15 breakpoint was located within the *HERC2P3* pseudogene, explaining the poor mappability in the short read WGS data, in contrast the chromosome X breakpoint was found within a nonrepetitive intergenic region. Upon manual inspection, the breakpoint was found in the short‐read WGS data of daughter 1 as well, supported by only one correctly mapped read pair (Figure 1d). Using the 10X genomics linked‐reads, we could pinpoint the breakpoint junction at the nucleotide level, revealing that the translocation occurred within a 6 nt long stretch of G (Supporting Information: Figure S1), the breakpoint junction is otherwise blunt and nonrepetitive.

Due to the unbalanced segregation of chromosomes 15 and X, both sisters have lost genetic material on chromosome 15p and chromosome Xp. The chromosome 15p deletion covers the entire p arm, the centromere, a small portion of proximal 15q, and total 21 Mbp including two pseudogenes, *CHEK2P2* and *HERC2P3*, not known to cause disease. The chromosome Xp deletion covers 1.5 Mbp, and includes eight genes, seven of which are unlikely to cause disease (*GTPBP6*, *PPP2R3B*, *PLCXD1*, *CRLF2*, *CSF2RA*, *IL3RA*, and *SLC25A6*). The deletion of *SHOX* resulted in both girls having symptoms concordant with Leri‐Weill dyschondrosteosis (MIM# 127300).

We analyzed publicly available data to determine if the translocation affected any TAD (Wang et al., 2018). Notably, the TADs differ across different tissues indicating technical or biological variability. In thymus, the translocation affects a TAD that includes the majority of Xp22.3. In contrast, there is no TAD located at the translocation breakpoint in cortex, small bowel, and lung (Supporting Information: Figure S2). In small bowel, the translocation affects the TAD covering PWCR, conversely the TADs appear fragmented in thymus, cortex, and lung, resulting in no perturbation of the TAD covering PWCR in those tissues (Supporting Information: Figure S3).

### 3.2 RNA sequencing of patient‐derived neural cells

**Figure 2:**
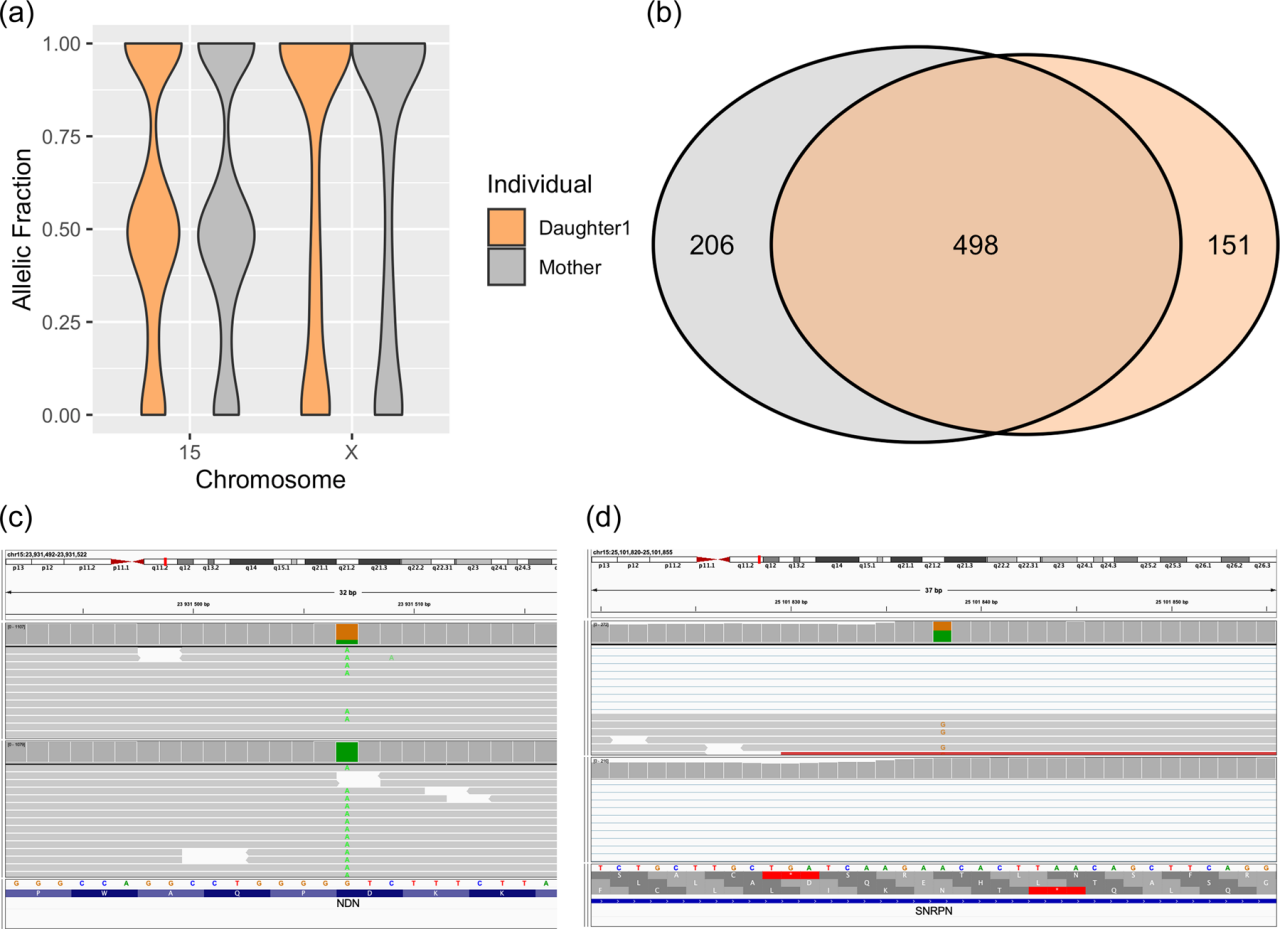
Allele specific expression across chromosome 15 and X. (a) The distribution of allelic expression in daughter 1 (orange), and her mother (gray). (b) Venn diagram detailing the number of shared SNV expressed on chromosome X. IGV screenshots illustrating biallelic expression of the paternally imprinted genes SRPN (c) and NDN (d), in daughter 1 (upper track), but not in her mother (lower track). IGV, Integrative Genomics Viewer; SNV, Single nucleotide variant.

SNV calling and allele‐specific expression analyses were performed on RNA‐seq data from neural stem cells derived from daughter 1 and the mother. Skewed‐X inactivation was found in both individuals, however, chromosome 15 was expressed in a biallelic manner (Figure 2a). Focusing on informative SNVs on the X chromosome, the daughter 1 cell line expressed 649 SNVs and the maternal cell line expressed 704 SNV. Comparing these SNVs, it was found that only 498 (58%) were shared, indicating that these individuals are not expressing the same X chromosome in the stem cell lines (Figure 2a). Follow‐up studies revealed no skewed X‐inactivation in blood or fibroblasts (data not shown), indicating that the observed skewed X inactivation represents a characteristic of the stem cells rather than a tissue specific behavior. Analysing the PWCR it was revealed that the daughter 1 cell line express *NDN* (Figure 2c), *SNRPN* (Figure 2d), and *SNHG14* in a biallelic fashion, in contrast the maternal cells displayed monoallelic expression of these genes. To validate these findings, we measured *SNRPN* expression using ddPCR of cDNA from fibroblasts in the mother and both daughters and the results were consistent with the RNA‐seq data (biallelic in daughter 1, and monoallelic in the mother as well as in daughter 2) (Supporting Information: Figure S4). The monoallelic *SNRPN* expression in daughter 2 confirms that the PWS region on the derivative X is leaky since no expression is expected in that sample.

**Figure 3:**
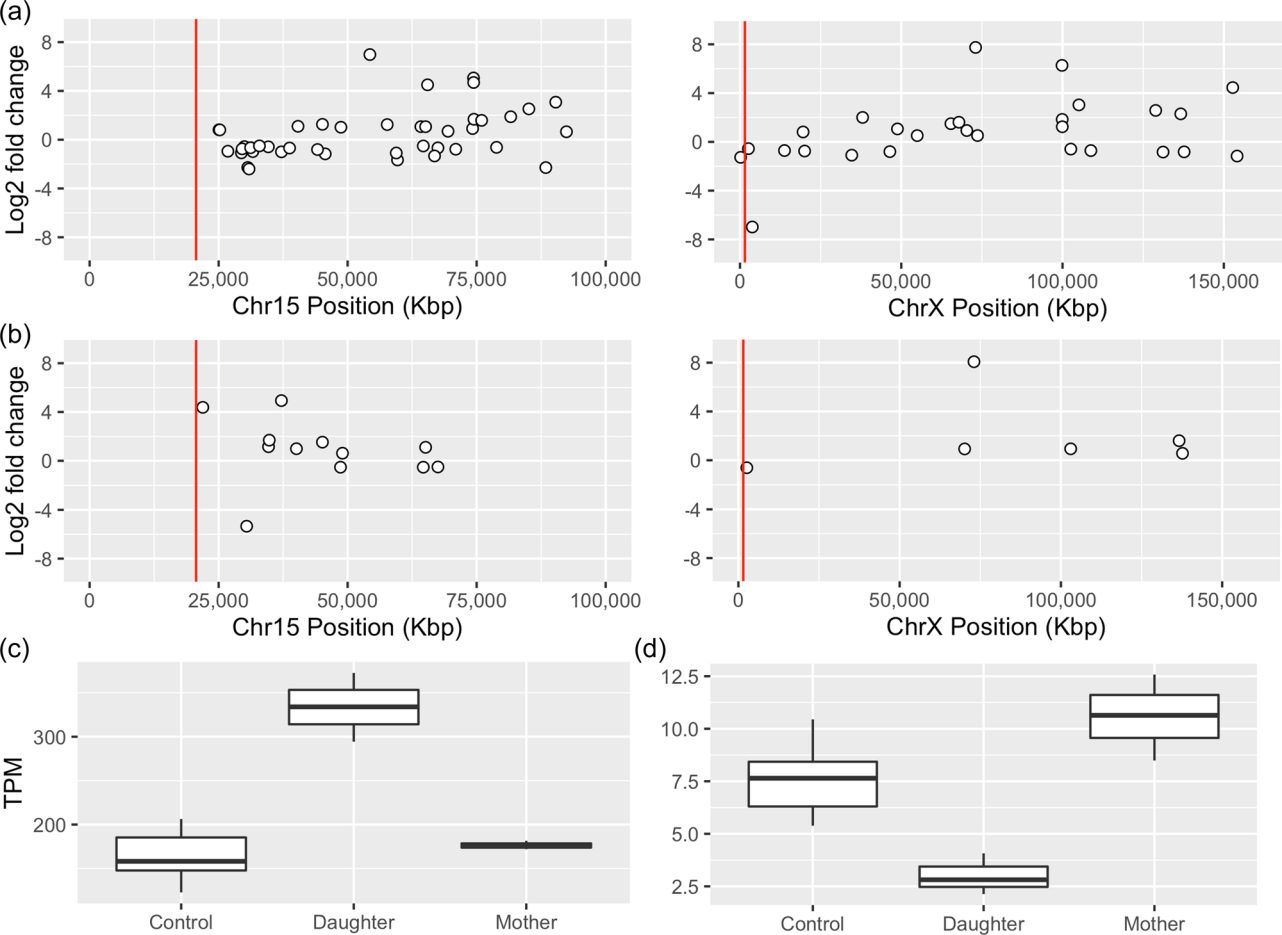
Differential expression analysis of daughter 1 and her mother. Differentially expressed genes on chromosome 15 and chromosome X in the daughter (a), and the mother (b). The log fold values are shown on the *Y*‐axis, and chromosomal position on the *X*‐axis, the red line indicate the breakpoint position of the t(15;X). (c) Boxplot illustrating the *SNRPN* expression levels in controls, daughter 1, and mother. (d) Boxplot illustrating the *PLCXD1* expression level in controls, daughter 1, and mother.

Next, we performed a differential expression analysis resulting in the discovery of 217 differentially expressed genes (DEG)s in the mother cells (Supporting Information: Table S1) and 911 DEGs in the daughter 1 cells (Supporting Information: Table S2) (Figure 3a,b). A biological function enrichment analysis shows that the DEGs of daughter 1 are enriched for pathways relevant to neurodevelopment, including *Neuronal projection* (*p* = 2.03E−03), *Neuronal cell body* (*p* = 1.29E−02), and *post‐synaptic density membrane* (2.33E−02) (Supporting Information: Table S3). In contrast, no such enrichment was observed in the mother (Supporting Information: Table S4). Daughter 1 carry 45 DEGs on chromosome 15 (Figure 3a), including two imprinted genes, *SNRPN* (Figure 3c) and *SNHG14*. The two genes overlap and are located 4.5 Mbp from the translocation breakpoint junction.

### 3.3 Multi‐omics analysis

Taking advantage of the linked read WGS phase blocks and informative SNVs from the RNA‐seq and WGS analysis in daughter 1, we could determine that the mother expresses *SNRPN* and *SNHG14* exclusively from the der(X)t(X;15). This finding indicates that t(X;15) originates from the maternal grandfather of daughter 1. To validate this finding, we performed a targeted analysis and searched for DEGs within the PWCR in the daughter 1 RNAseq data, resulting in the discovery of 11 DEGs including the genes *GABRB3*, *KLF13*, and *TJP1* (Supporting Information: Table S1; Figure 3a).

Altogether, daughter 1 and her mother carry 27 and 7 DEGs, respectively, on chromosome X, representing 2% of the DEGs of each individual. Searching within the deleted region on chromosome X, one gene located in the PAR1 region (*PLCXD*1) is downregulated compared to controls in daughter 1 but not in the mother (Figure 3d). *SHOX* was not expressed in any of the cell lines analyzed by RNA‐seq (controls, daughter 1, or mother), and could therefore not be assessed. Interestingly, both mother and daughter show significant upregulation of the *XIST* gene.

**Figure 4:**
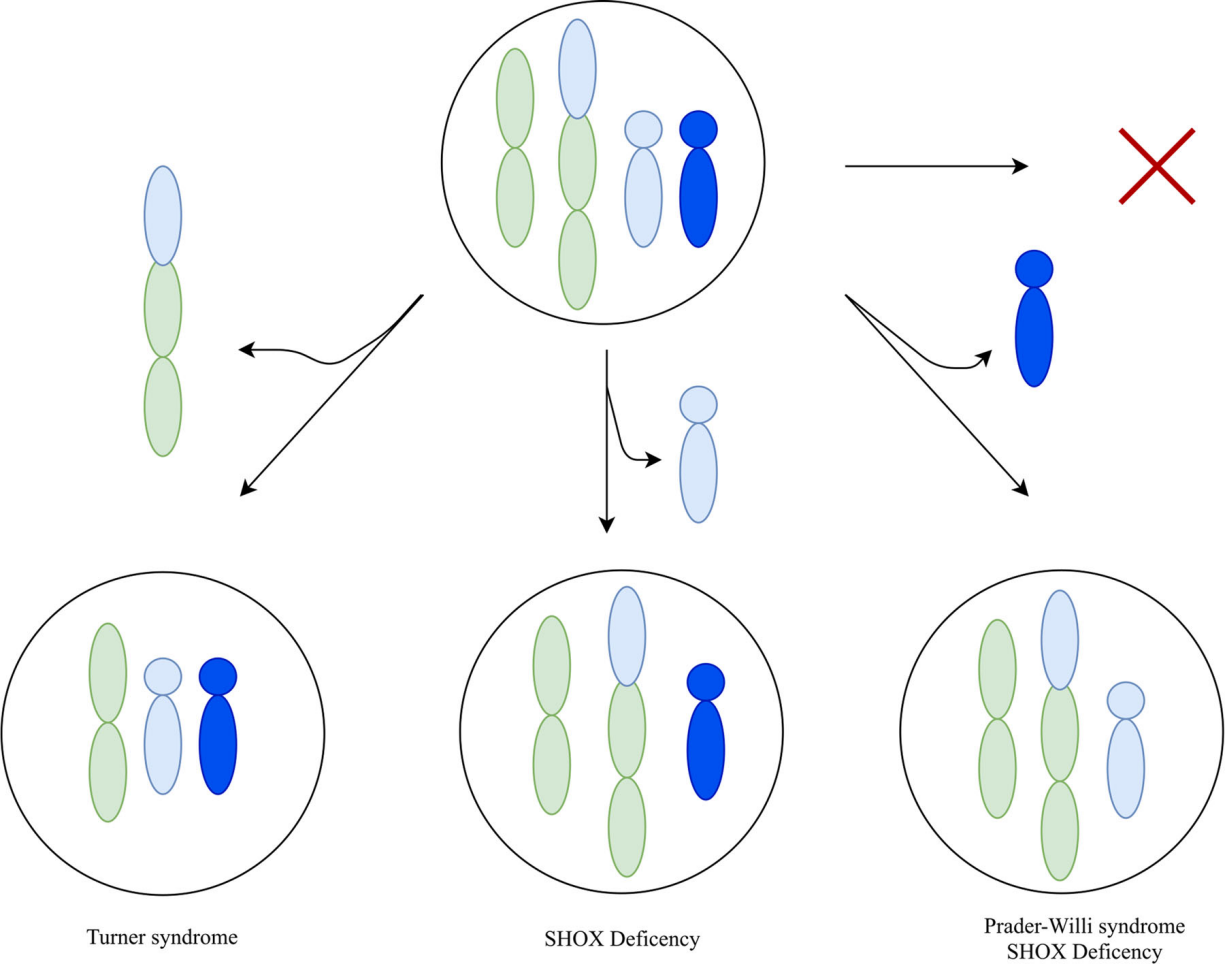
A schematic illustrating possible outcomes of trisomic rescue triggered by unbalanced segregation of t(X;15). Each circle represents a cell. The colored objects represent chromosomes: green for chromosome X, green and blue the t(X;15) derivative chromosome, light blue maternal chromosome 15, and dark blue paternal chromosome 15. Each pathway indicates the elimination of one of the extra copies of chromosome 15. The red cross indicates no elimination of chromosome 15, which is not compatible with life.

Our combined results indicate that the phenotypes are a result of trisomy rescue of chromosome 15 after an unbalanced segregation of the derivative chromosomes (Figure 4). In particular, we conclude that daughter 2 present a methylation pattern consistent with a loss of the paternal chromosome 15. In contrast, daughter 1 carry a normal copy of paternal chromosome 15, as well as the maternal der(X) chromosome, indicating that the maternal chromosome 15 was discarded.

### 3.4 Droplet digital PCR

The biallelic expression of *SNRPN* observed in transcriptome data from NES cells derived from daughter 1 was followed by ddPCR of cDNA from fibroblasts. Analysis of cells obtained from the mother, daughter 1, and daughter 2 were analyzed and showed monoallelic expression in the mother and daughter 2, and biallelic expression in daughter 1 (Supporting Information: Figure S4).

## 4 DISCUSSION

Here, we provide a unique description of two sisters, affected by two different complex conditions caused by unbalanced segregation of an X;15 translocation, followed by trisomy rescue of chromosome 15 (Figure 4). This resulted in one sister with PWS and Leri‐Weill dyschondrosteosis (daughter 2) and the other sister also with Leri‐Weill dyschondrosteosis as well as a complex PWS‐like phenotype (daughter 1).

The phenotype observed in daughter 1 overlaps with that of PWS, including obesity and intellectual disability. However, she does not have maternal UPD15, nor does she display any methylation defects. The biallelic expression and upregulation of *SNRPN* observed in daughter 1 highlights that the PWCR is indeed affected by the translocation.

Specifically, it appears as if the imprinting of *SNRPN* is less stringent resulting in abnormal biallelic expression. This phenomenon may be due to the translocation causing perturbation of TADs (Supporting Information: Figures S2 and S3), or by positioning a powerful enhancer closer to the *SNRPN* promoter site.

The mother does not exhibit upregulation of *SNRPN*, and expresses *SNRPN* from a single allele only, namely from der(X). From these patterns, and the phasing of der(X), we conclude that der(X) is of paternal origin (i.e., the maternal grandfather of daughter 1), and therefore, the leaky imprinting will not be visible.

PWS is caused by the loss of expression of the paternally imprinted genes in PWCR, however, in daughter 1, we discover upregulation of such genes, including *NDN* and *SNRPN*. Interestingly, it has been shown that both up and downregulation of *SNRPN* may disturb brain development (Li et al., 2016); conversely, we discover downregulation of *GABRB3*; which is consistent with PWS (Butler, 2011). As such, we hypothesize that the X;15 translocation perturbs the expression of several genes within PWCRN, either directly or indirectly, explaining the PWS‐like phenotype of daughter 1.

Daughter 2 is affected by maternal UPD, she is carrying the t(X;15) as well as the unaffected maternal chromosome 15 (Figure 4). As such one could hypothesize that the leaky expression of der(X)t(X;15) is advantageous to daughter 2, as it allows her to express paternally imprinted genes in the PWCR, which could result in a milder clinical presentation (Supporting Information: Figure S4).

In the RNA‐seq data, cells from both daughter 1 and the mother express chromosome X in a skewed‐fashion (Figure 2), however, we find no skewed X inactivation in blood. It is known that the X‐inactivation is maintained after re‐differentiation of fibroblasts (Liu et al., 2010); as such, these patterns could indicate tissue specific X‐inactivation in fibroblasts, or some growth artefact, providing an advantage to cells expressing a certain X chromatid. Either way, we argue that the X inactivation is of minor importance, since these individuals express different X chromosomes, seemingly without affecting the expression of chromosome 15.

## 5 CONCLUSIONS

In conclusion, we present a unique pedigree, consisting of a healthy mother and her two daughters, affected by combinatory phenotypes. Both daughters suffer from *SHOX* deficiency, as well as varying degrees of PWS. These phenotypes are explained by an X;15 translocation and trisomy 15 rescue, resulting in PWS due to maternal UPD15 in one sister and Prader–Willi like syndrome due to leaky imprinting of PWCR in the other sister. Our analyses allowed us to phase the derivative chromosomes and revealed that genes in the PWCR are expressed partially in a biallaleic manner. In short, a multi‐omics approach was necessary to solve this unique familial rearrangement and a novel mechanism causing PWS through long‐range interactions was uncovered.

## CONFLICT OF INTEREST

A. L. received honoraria from Illumina. The remaining authors declare no conflict of interest.

## Supporting information

Supporting information.

Supporting information.
