# Supplementary material for: Multi‐omics analysis reveals multiple mechanisms causing Prader–Willi like syndrome in a family with a X;15 translocation
Source: Hum Mutat. 2022 Jul 23;43(11):1567–75. doi: 10.1002/humu.24440 (PMC9796698; doi:10.1002/humu.24440)

**Figure S1.** Translocation breakpoint junction analysis


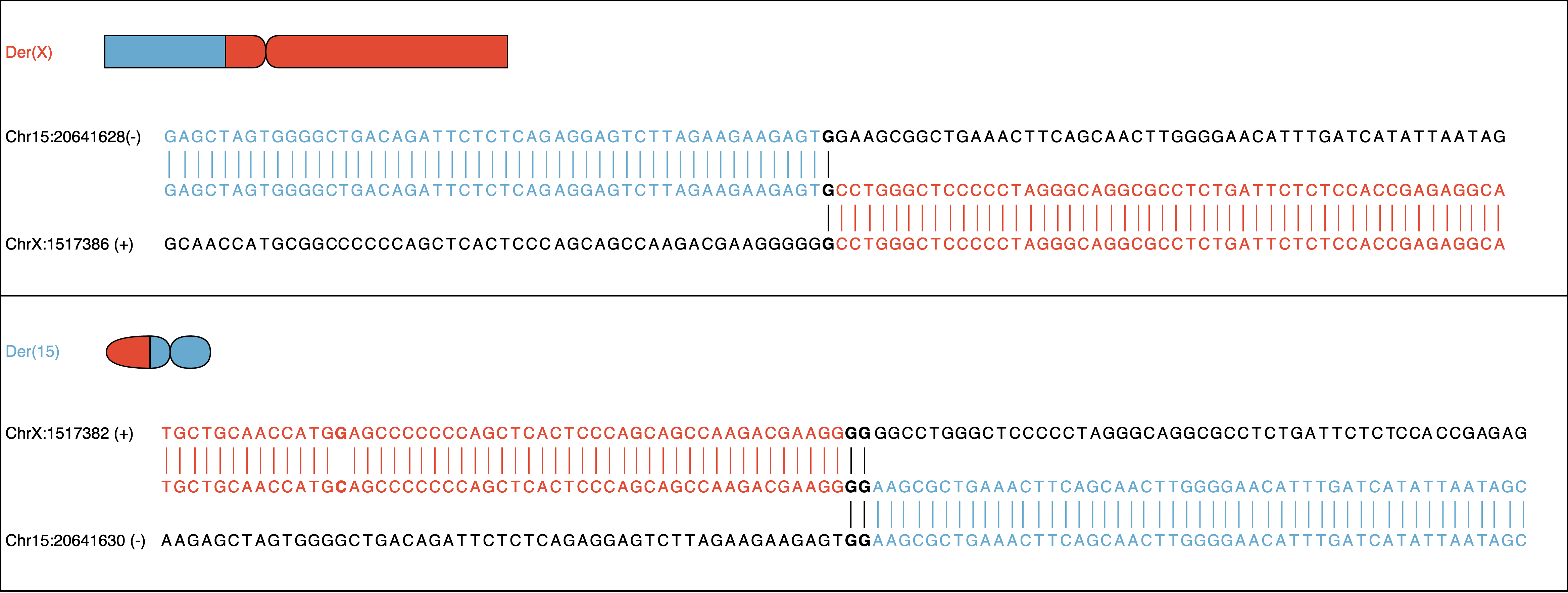


**Figure S2.** Topologically associated domains (TADs) surrounding the genomic breakpoint on chromosome X. Each pane indicates TADS in a specific tissue (Thymus, Cortex, Lung, and small bowel).


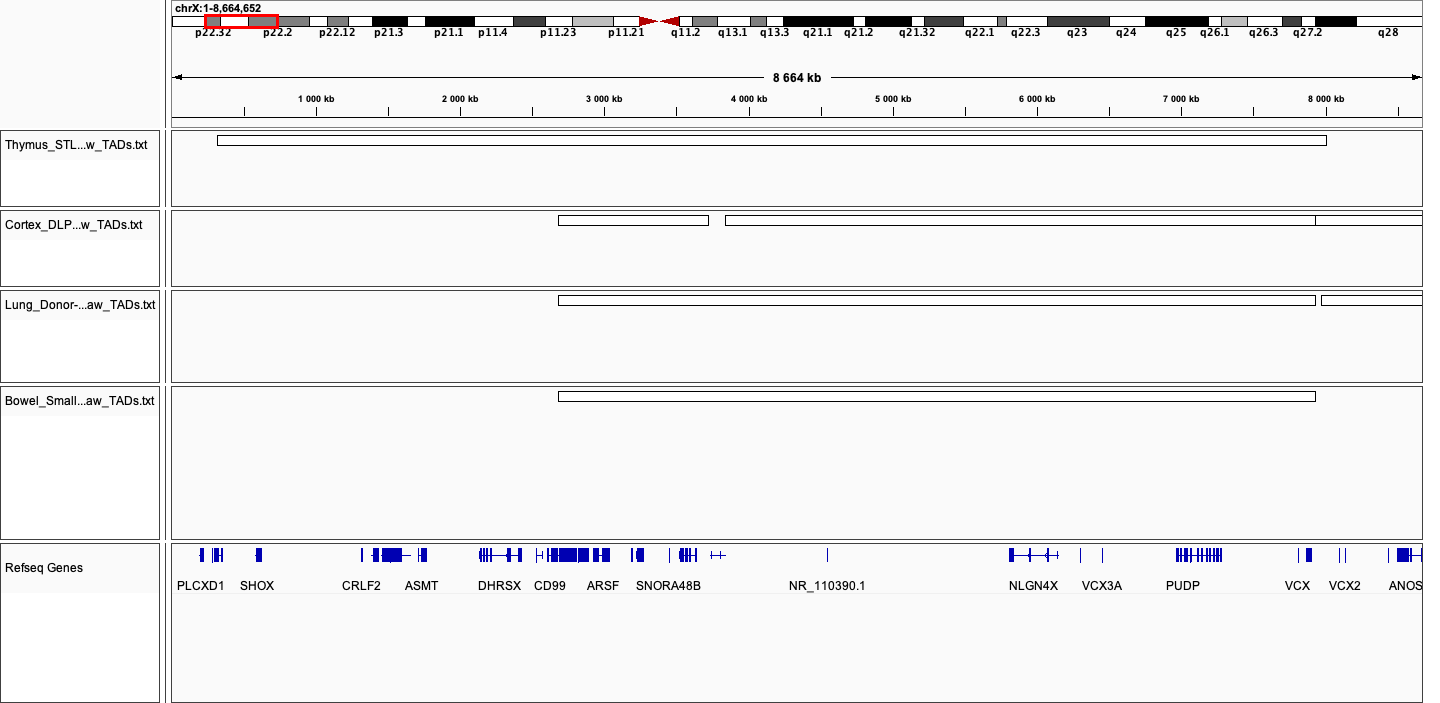


**Figure S3.** Topologically associated domains (TADs) surounding the genomic breakpoint on chromosome 15. Each pane indicates TADS in a specific tissue (Thymus, Cortex, Lung, and small bowel)


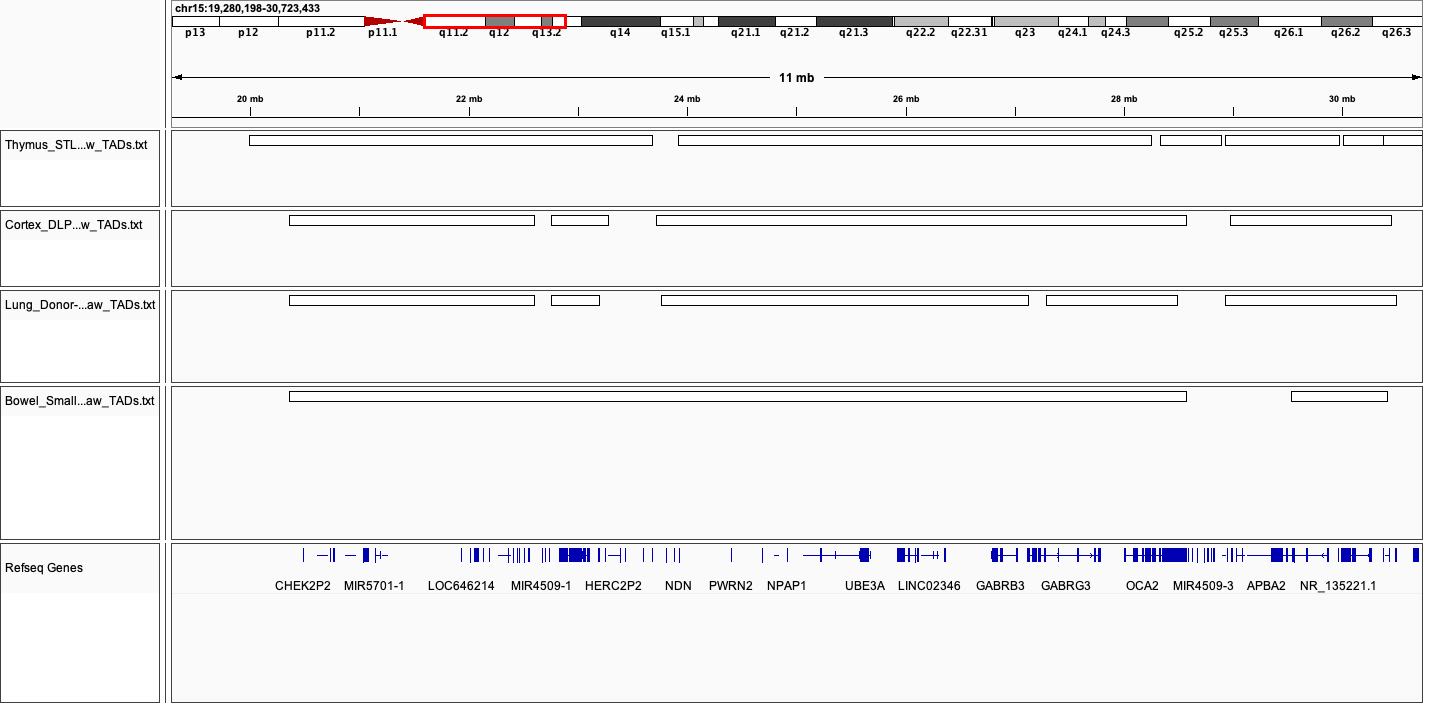


**Figure S4**. ddPCR results showing mono-allelic expression in the mother and daughter 2 and bi-allelic expression in daughter 1.


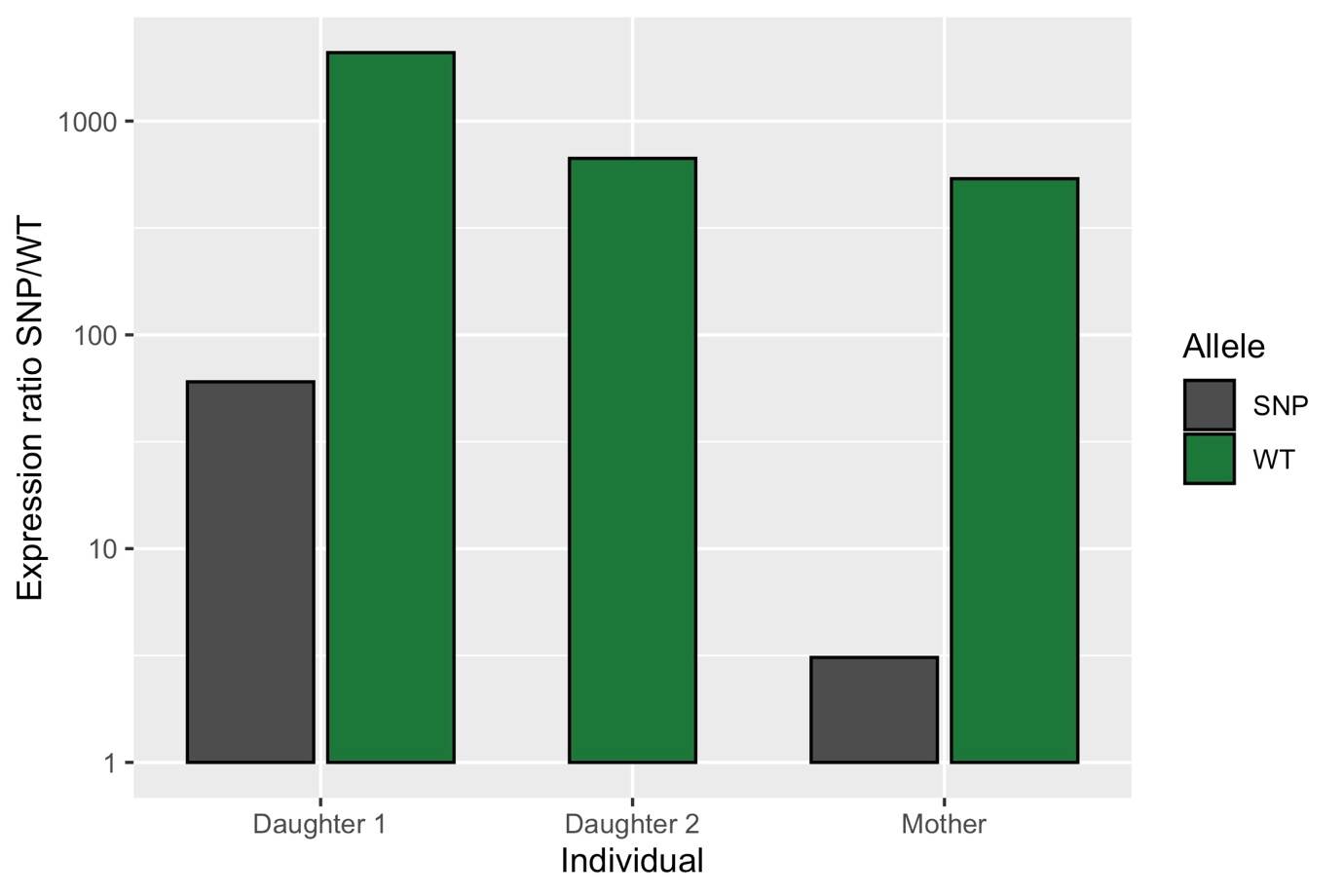

Supplement: Supplementary file 1 — Supporting information. [file HUMU-43-1567-s001.docx]
